# Using theory to interpret how senior clinicians define, learn, and teach clinical reasoning

**DOI:** 10.15694/mep.2017.000182

**Published:** 2017-10-12

**Authors:** Waverley Gee, Megan Anakin, Ralph Pinnock

**Affiliations:** 1 University of Otago

**Keywords:** clinical reasoning, dual process theory, script theory, teaching methods

## Abstract

This article was migrated. The article was marked as recommended.

**Introduction**: Dual process theory and script theory have been used to understand and explain how students learn clinical reasoning. This study used these tools to interpret how experienced teachers described their definitions of clinical reasoning, their own history learning about clinical reasoning, and their methods of teaching clinical reasoning.

**Methods**: Interview data from 14 senior clinicians were investigated thematically using a general inductive approach then interpreted using principles and concepts associated with dual process theory and script theory, including the concept of deliberate practice and script consciousness.

**Results and Discussion**: Senior clinicians’ definitions of clinical reasoning were consistent with the literature. Few of them recalled being explicitly taught clinical reasoning. They identified teaching as a way to further develop their own clinical reasoning. They taught it opportunistically using an apprenticeship and role-modelling approach in clinical contexts. Their teaching techniques included supervised practice, reflection, think aloud, focused data collection guided by the clinical presentation, and iterative reasoning.

**Conclusion**: Dual process theory, script theory, and the concepts of mindful practice and deliberate practice were found to be useful tools to understand how senior clinicians taught and learned clinical reasoning. These findings are guiding our clinical reasoning curriculum and faculty development programme.

## Introduction

Clinical reasoning has been described as the ability of physicians to become efficient diagnosticians ( Eva 2005, Trowbridge et al 2015). This thinking process has been partially explained using dual process theory from the field of cognitive psychology ( Kahneman & Tversky 1982). In brief, dual process theory is described by two competing thinking processes characterised as fast and slow. The fast process is regarded as non-analytical and spontaneous, whereas, the slow process is analytical, deliberate, and underpins our hypothetical-deductive reasoning ability. Dual process theory has been used to identify students’ diagnostic difficulties and suggested possible remediation techniques such as teaching them about the possible sources of errors and biases in their clinical reasoning ( Audétat et al 2017, Norman et al 2017). Dual process theory appears to be useful when examining students’ challenges when learning clinical reasoning. The concept of ‘mindful practice’ has been suggested to address diagnostic errors (Crosskerry 2013). ‘Mindful practice’ involves becoming aware of biases in our thinking. Biases can include ‘search satisficing’ where other possible causes are rule out too early or without sufficient information, and ‘limited framing’ where prior experiences may restrict the possible diagnoses considered.

Clinical reasoning has also been theorised using the concept of ‘illness scripts’ ( Charlin et al. 2000, Schmit & Rikers 2007). To enable medical students to develop expertise as diagnosticians, script theory suggests that “the acquisition of medical expertise consists in building, refining, and linking scripts that allow students to become active processors of clinical information instead of simple collectors of as much information as they can get, without giving meaning to it” ( Charlin et al. 2000, p. 189). Instructional techniques have been proposed to assist students in the active construction of illness scripts. These techniques include providing exposure to real patients in clinical settings that is underpinned by sound biomedical knowledge ( Charlin et al. 2000), using examples to model the flexible and context-specific integration of analytic and non-analytic thinking approaches ( Eva 2005), and promoting reflection and further elaboration about the clinical problems students experience ( Schmit & Rikers 2007). To account for the changing learning needs of students, a developmental teaching sequence has been proposed featuring ‘whole-case’ and ‘serial-cue’ approaches ( Schmidt & Mamede 2015). At the start of their education, Schmidt and Mamede recommend that students are supported with a ‘whole case’ approach where they are provided with all of the information and then seek to identify features that support a likely diagnosis. Once students have constructed general illness scripts for common presentations, it is recommended that they are exposed to a ‘serial-cue’ approach. This approach mimics clinical practice in which clinical information is analysed during history taking. ‘Self-explanation’, ‘enabling conditions’ and ‘deliberate reflection’ have been suggested to enhance students’ construction of illness scripts. ‘Self-explanation’ involves a student explaining how a patient’s signs and symptoms are related to possible diagnoses ( Chamberland et al. 2011, Chi et al. 1994). ‘Enabling conditions’ refers to “knowledge of the conditions under which disease manifests” ( Schmidt & Mamede 2015 p. 969). Teachers can use ‘enabling conditions’ as a gauge for assessing how detailed a student’s illness script for a particular disease may be. ‘Deliberate reflection’ allows students to further develop their illness scripts by comparing and contrasting the features they identified in the patient’s history to support or challenge their initial diagnosis ( Mamede et al. 2012). From this brief survey of select literature, script theory may be a useful resource to help us understand how to teach clinical reasoning to medical students.

In clinical learning settings, however, “script building remains an individual exercise influenced by idiosyncratic experience” ( Lubarsky et at 2015, e68). Consequently, Lubarsky and colleagues have suggested the term ‘script consciousness’ to describe how a teacher’s explicit use of illness scripts may enhance the learning process for students. To date, however, there has been no research evidence published using the concept of script consciousness as an analytic tool to interpret how experienced teachers describe their definitions of clinical reasoning, their own history learning about clinical reasoning, and their methods of teaching clinical reasoning.

There is limited literature on how experienced clinicians develop, maintain, and assess their clinical reasoning. In general, clinicians reported that they maintained their skills via peer-review in clinical practice and by teaching undergraduate or postgraduate students ( Durning et al 2013). To increase our knowledge about how clinicians understand and view their learning and teaching of clinical reasoning, we aimed to apply principles and concepts from dual process theory and script theory to the descriptions of clinical reasoning of senior clinicians teaching at our medical school.

## Methods

### Context of the study

This study took place at the University of Otago where the medical curriculum for undergraduate students is taught in two parts: Early Learning in Medicine (ELM) and Advanced Learning in Medicine (ALM). In ELM, students receive case-based teaching in small groups and practise the Calgary-Cambridge method of history taking (Kurtz et al. 2003) with simulated patients. ELM emphasises foundation medical knowledge in the context of skills including the principles of clinical reasoning. These principles include how disease prevalence influences diagnosis, common errors and biases, type I (fast) and type II (slow) thinking, Bayes’ theorem, and the sensitivity and specificity of investigations. Students are also taught the value of reflection-in-action and reflection-on-action (Schön 1983) for thinking during and after diagnosis, respectively.

In ALM, students’ learning experiences are predominantly clinical. Teaching occurs informally in workplace settings and, therefore it is largely unstructured but responsive to events that occur with real patients during clinical rotations. There is a risk that students may experience the ad hoc teaching in ALM as disconnected from their previous learning in ELM because it lacks a formal and explicit structure. We know that our students are given the opportunity to learn clinical reasoning on their rotations, however, exactly what they are learning or how it is being taught is unclear. Most our senior clinicians are from a generation that when they were undergraduates, they were unlikely to have been formally taught clinical reasoning. How their own education might influence their teaching was unknown prior to this study.

### Study design and participants

Convenience sampling was used to recruit senior clinicians to participate in this qualitative interview study. A senior clinician was defined as consultant physician affiliated with Dunedin School of Medicine, who supervised and taught students in ELM and ALM settings. The senior clinicians invited to participate in the study were chosen because they were identified as experts in clinical reasoning based on their seniority and the opinion of their peers. We actively recruited participants for 6 weeks via email and by approaching potential participants directly.

Fourteen senior clinicians consented to participate. Twelve participants were male and two were female. All participants identified themselves as New Zealanders of European descent. Participants had 20-45 years of experience as physicians. Their involvement with teaching matched their experience as physicians; they all spoke of how they were required to teach more junior colleagues from the time they were given roles such as house officer or registrar. Only one participant had a university qualification in teaching and it was a postgraduate certificate in clinical education. Participants had expertise in cardiology, gastroenterology, general practice and rural health, neurology, obstetrics and gynaecology, paediatrics and child health, respiratory, or rheumatology.

### Ethics approval

Ethics approval for this study was granted by the Human Ethics Committee at the University of Otago (Ref: D16/295). The Ngai Tahu Research Consultation Committee was consulted. Participants were welcome to withdraw from the study at any time and received no incentive for participating.

### Interview questions

A set of questions was developed to guide the semi-structured interviews, since there were no existing validated questionnaires to assess how clinical reasoning is taught. The questions were based on current understandings of how clinical reasoning is taught and learned in medical education (e.g., Schmidt & Mamede 2015). Two experienced researchers in medical education, who were external to the research team, reviewed the questions and provided helpful comments. The questions related to three main topics: how they define clinical reasoning, how they learned clinical reasoning as students and maintain their skills as teachers, and how they teach clinical reasoning to their own students.

### Data collection and analysis

An iterative approach was used to collect and analyse the data. Data collected from earlier interviews were analysed and used to inform the data collection in subsequent interviews. Two of the authors (WG & MA) conducted semi-structured interviews with each senior clinician in November or December 2016. Interviews ranged from 20-30 minutes in length. As relative outsiders to the medical profession with some knowledge of clinical reasoning, the interviewers were not expected to have an undue influence on the information gathered from the participants. Each interview was audio recorded, then transcribed verbatim and de-identified. The other author (RP), an experienced senior clinician, debriefed the interviewers and participated in the analysis of the data. This approach also allowed for a better appreciation of the patterns present in the data, with both outsider and insider perspectives on analysis.

The interview data were analysed independently by each of the authors using a general inductive approach (Thomas, 2006) and script theory ( Charlin et al. 2000, Schmit & Rikers 2007). Themes relating to the three main study topics were identified in each of the transcripts. These themes were organised using the qualitative research analysis tool, HyperResearch (ResearchWare, 2015). The authors then met to compare themes and discuss their interpretation using principles and concepts from dual process theory and script theory as findings.

## Results

### Definitions of Clinical Reasoning

Before interpreting the data theoretically, it was important to understand how participants described their definitions of clinical reasoning. When asked to define clinical reasoning, senior clinicians’ initial responses were “I never thought about a definition before” (P13) and “I never looked this up formally, to be quite honest, so I have to speculate what it could mean for me” (P6). However, once they were prompted to reflect on their practice, their definitions and understandings of clinical reasoning were remarkably similar and consonant with definitions reported in the literature (e.g., Eva, 2005, Gay et al., 2013).

Well I suppose clinical reasoning is the ability to synthesise the information that you’ve obtained from the history, examination, and investigation, and formulate it into a series of problems and diagnoses. (P12)

I would probably define clinical reasoning as the cognitive processes that we go through or that we use to, primarily to reach a diagnosis. But also to address therapy, treatment. (P7)

These senior clinicians expressed awareness that a physician had to activate and use their medical knowledge to make sense of diagnostic information; however, they did not make explicit reference to illness scripts or any other theoretical concepts to describe the clinical reasoning process they were defining.

How senior clinicians learned clinical reasoning and maintain their skills

Participants could not recall any explicit teaching of clinical reasoning. “Oh I wasn’t taught clinical reasoning at all, I learnt it by osmosis” (P1). If they made connections to their medical training, they discussed learning clinical reasoning from discussions with colleagues and mentors about patients during their clinical rotations, “I think I probably just picked it up, what rudimentary skills I have, from working with others” (P3) and “seeing how other people go about it. You know, examples of your senior people you work with” (P8).

When asked how they maintained their clinical reasoning skills, only two senior clinicians thought their teaching had changed little or not at all, whereas, others noted changes in their approaches or attitude towards teaching.

I didn’t know I was developing my teaching, I’m just trying. I haven’t, it hasn’t been a particular process, just a journey. So hopefully now, certain aspects of how I teach are more sophisticated and more effective and efficient from what I was doing ten years ago or fifteen years ago. (P2)

I was able to feel relaxed enough to expose my thinking to other people. Because the problem with that is, if you make a mistake, everyone knows. And as I’ve got older, that bothers me less and less. (P1)

The guidance given to senior clinicians when they were students was tacit rather than explicit. As learners, they were expected to acquire their skills through observing more senior colleagues in practice. Now as teachers, they continued to develop their skills informally, tacitly, and idiosyncratically. The descriptions of these learning experiences were consistent with their definitions of clinical reasoning; again, they did not make explicit reference to illness scripts or any other theoretical concepts to describe the clinical reasoning process they had learned.

### How senior clinicians teach clinical reasoning

When senior clinicians described their teaching methods, their descriptions involved role-modelling with a think aloud technique ( Pinnock et al, 2016).

I would always try and explain why I’ve reached that conclusion, it’s not some magical ability that we have as a doctor or as a consultant or whatever, rather than you’ve been here before and you can kind of read the signals appropriately. (P3)

I try to lead by example there but also try to emphasise certain things that the students need to look out for. (P6)

Senior clinicians expressed that much of their reasoning is automatic and unconscious. “I’m quite a fast thinker, but I have structures and processes in terms of what I do.. I try to model my thinking when I justify a diagnosis” (P2). To slow down and make their thinking explicit, one senior clinician used “the think aloud approach asking them as we are going through the history what they are thinking and explaining to them what I am thinking” (P13).

Senior clinicians felt students required opportunities to articulate their thinking about their clinical experiences under supervision with feedback. The use of the word ‘experience’ was interpreted to mean practice. For example, “..having experience counts for a huge amount so presenting a case and then being asked ‘what do you think it is and why?’” (P2).

Rather than discussing how they taught specific medical knowledge, senior clinicians made reference to its importance when teaching clinical reasoning, “knowledge is better retained when it is part of clinical reasoning” (P9).

When theory was used to interpret these teaching techniques, six of teaching method described by senior clinicians corresponded to instructional techniques proposed to assist students in the active construction of illness scrips ( Charlin et al. 2000, Eva 2005, Schmit & Rikers 2007) and the reminder corresponded to mindful practice underpinned by dual process theory ( Croskerry 2013). As a group, our participants described nine aspects of clinical reasoning that they taught using a serial-cue approach in clinical contexts. These aspects are presented as learning points roughly in the order they described by the participants (see Figure 1).

**Figure 1.** Learning points emphasised by senior clinicians when teaching clinical reasoning to their students. Note * denotes correspondence with dual process theory ( Kahneman & Tversky 1982), whereas, the remainder correspond to script theory ( Charlin et al. 2000, Schmit & Rikers 2007).

Learning point	Representative extracts from senior clinicians
Questioning with possibilities in mind*	*Think of a differential diagnosis right from the beginning* (P7) *Tailor your history and examination to the patient’s problem* (P12)
Recognising the pitfalls of fast thinking*	*the other thing that can happen over the years is that you start jumping to conclusions because you think you seen it so often before - there is the danger as you get older you just make so many assumptions* (P11) *Students have read about a case - they tend to jump to conclusions* (P7)
Analysing while collecting information	*Consider coming up with a diagnosis, make some priorities... think about what is common. What should not be missed?* (P8) *Think of options early, don’t leave it till the end* (P3)
Reviewing possible diagnoses iteratively	*You are continually coming back and reviewing the diagnosis* (P2) *I tell my students also that going back to the history while you’re performing the physical exam is important because some things might pop up that the patient hasn’t mentioned. Surgical scars for instance, the patient’s forgotten* (P6)
Starting broadly then narrowing down*	*Well one of the trickiest things is this balance between ... having a wide lot of possibilities and narrowing down on some of them. And how soon you should do that narrowing down as opposed to keeping it open. So one of my little pet hates is the word ‘rule out.’* (P9)
Prioritising based on prevalence	*We do often talk about the likelihood of a condition* (P4) *If you hear hooves you’ve got to think horses not zebras.* (P5)
Managing uncertainty	*Making sure [students] are aware of the uncertainty of clinical medicine. That actually not knowing is perfectly fine, because there is a difference between not knowing and getting it wrong* (P5)
Encouraging case-based discussions	*The students read the cases and then in groups they work through a series of guided questions so that they’re encouraged to think about what features of the history are important, what’s important to the patient, what investigations might need to be done, what treatment might be important. (P12)* *Having case discussions where they bring a case and in groups we discuss their cases in a variety of ways. (P14)*
Modelling metacognitive awareness	*How you consciously review your thinking is important* (P10) *We need to get into the habit to think about our thinking* (P9)

## Discussion

It appears that senior clinicians developed their approach to teaching clinical reasoning through their own informal and idiosyncratic learning opportunities in clinical settings. Even though they continue to use an apprenticeship model to teach clinical reasoning to their students, the learning points they emphasised are consonant with the recommendations in the medical education literature underpinned by dual process theory (eg. Audétat et al 2017, Norman et al 2017) and script theory (eg. Lubarsky et at 2015, Schmidt & Mamede 2015). Senior clinicians’ learning points may be interpreted to be script-oriented and developed from mindful practice rather than originating from a script consciousness perspective.

First, not all of the senior clinicians interviewed were familiar with the term clinical reasoning and could not give a precise definition of the term. On reflection, they demonstrated an understanding consistent with the term’s use in medical education literature (eg. Eva 2005, Trowbridge et al 2015). They described clinical reasoning as the thinking process they used to arrive at possible solutions to a patient’s presentation. This description appeared to be sufficient for them to teach clinical reasoning to their students.

Second, senior clinicians modelled their teaching on selected interactions with their own teachers and drew upon a varied repertoire of knowledge and prior clinical experiences. In common with other tertiary education teachers, they continue to use these informal learning opportunities to compensate for their lack of formal teaching instead of “teaching the way they were taught” ( Oleson & Hora 2014, p. 29). Additionally, and as noted by (Cox 2013), they reflect on their own learning and experience to emphasise particular aspects of clinical reasoning to promote student-centred learning opportunities for their own students.

Third, senior clinicians appeared to teach clinical reasoning with techniques that were script-oriented without making explicit reference to script theory or corresponded mindful practice to address errors and biases that are explained by dual process theory. For example ‘recognising the pitfalls of fast thinking’ was interpreted to make a direct reference to a principle of dual process theory ( Audétat et al 2017, Norman et al 2017). Other learning points were interpreted from a script theory perspective to provide insights into how illness scripts can become elaborated and interconnected. For example, ‘starting broadly and narrowing down’ was interpreted to articulate how students can learn to differentiate between illness scripts that may share characteristics. This learning point may also be interpreted as how students can learn to signpost the structure and organisation of their clinical knowledge in their illness scripts. By ‘encouraging case-based discussions’, senior clinicians may be tacitly helping students learn to use self-explanation ( Chamberland et al. 2011, Chi et al. 1994), identify enabling conditions ( Schmidt & Mamede 2015) and practice deliberate reflection ( Mamede et al. 2012).

Additionally, all of the learning points involved providing students with feedback as they assessed patients. For example, senior clinicians used ‘questioning with possibilities in mind’ and ‘modelling metacognitive awareness to encourage students to compare and contrast possible diagnoses and reflect on their thinking ( Mamede et al. 2014). Likewise, senior clinicians discussed ‘reviewing possible diagnoses iteratively’ to show how possible diagnoses may need to be revised as new information becomes available ( Eva 2005). Learning the importance of ‘recognising the pitfalls of fast thinking’ and ‘prioritising base on prevalence’ are two contrasting approaches that required students to move backwards and forward through the information they have gathered and reviewing it to pursue various possibilities ( Schmidt & Mamede 2015).

### Implications, limitations, and future directions

The findings from this study will be of interest to clinicians with teaching duties so they can make explicit connections between the teaching techniques they may already be using and current theory used to explain clinical reasoning for themselves and their students. In this way, teachers can demonstrate ‘script consciousness’ which has been proposed as a way to enhance the teaching of clinical reasoning ( Lubarsky et al 2015). Findings can also be used to further develop our use of theory in practice. Not only are senior clinicians interested in helping students to develop robust illness scripts and avoid cognitive errors and biases but they also appear to be investing time in modelling how illness script can be structured, organised, elaborated, and become interconnected. The role of feedback was central to all learning points described by the participants. Specifically, feedback appears to be used by teachers to help their students make their conversations with one another recognisable as clinical reasoning. The power of feedback to promote students’ reflection-in-action and reflection-on-action is well documented (Schön 1983) and is associated with gains in student learning (Hattie, 2008).

Despite the use of specific teaching strategies by our senior clinicians, our students appear to be learning clinical reasoning implicitly. To enhance the teaching of clinical reasoning, the concept of script consciousness could be introduced to teachers and students through small group discussion groups and case presentations. This explicit use of script theory would allow us to develop a shared language to describe the process of learning clinical reasoning for everyone to use at our institution.

Even though we found support in the literature for how we interpreted the results, our findings are limited to a single institution and a small number of experienced teachers. Missing from our findings are teaching techniques described by junior clinicians and clinicians who teach outside of a hospital setting.

Future studies could to explore how clinical reasoning is taught in other medical schools and be designed to include clinicians in more varied work settings and at different phases of their teaching career. We have begun to map stages in the development of students’ clinical reasoning (Pinnock et al in press), therefore, refining interview questions to target particular student characteristics may be a fruitful avenue to pursue.

## Conclusion

Our study adds to the growing literature supporting a theory-informed evidence-base for teaching clinical reasoning. We used principles and concept from dual process theory and script theory to interrogate interview data from experienced teachers at our medical school. We found that their descriptions of how they defined, learnt, and taught clinical reasoning could be interpreted productively using script theory as an analytical tool, predominantly. We found that our teachers were actively assisting students to construct illness scripts and become aware of their cognitive processing errors and biases. They were encouraging their students to understand how they were structuring and organising their illness scripts, as well as how their scripts could become elaborated, interconnected, and differentiated from one another. However, our teachers did not describe their teaching methods in terms of dual process theory or script theory. Instead, their teaching techniques were developed tacitly, informally, and idiosyncratically. Next steps will be to assist our teachers to formalise their teaching of clinical reasoning, by enhancing the apprenticeship model they use. Our participants viewed clinical reasoning as central to their practice and were committed to improving their teaching of it. As staff developers, our next step will be to help them formalise their teaching of clinical reasoning by introducing the concepts of mindful practice and script consciousness, and developing shared language to discuss clinical presentations and problems with one another and our students.

## Take Home Messages

• Teachers use an apprenticeship model to teach clinical reasoning on rotations.
• Teachers developed their teaching methods through experience and peer-review in the workplace.
• The core of their teaching is practice under supervision with feedback.
• They use cue-based teaching techniques that closely mimic clinical practice.
• They role-model clinical reasoning by making their reasoning explicit.
• Teachers instructional techniques can be explained using concepts from dual process theory and script theory.

## Notes On Contributors

Waverley Gee is a medical student now in her clinical years at the University of Otago. Her research interests include medical education and clinical reasoning. She is currently an education representative for her medical school council, contributing to the improvement of the course.

Megan Anakin is a Lecturer in Medical Education and Education Advisor at the University of Otago. She is also a teacher with over 25 years of experience in primary, secondary, and tertiary classrooms. Her research interests include conceptual development, faculty development, and how clinical reasoning is taught and learned by healthcare professionals.

Ralph Pinnock is a paediatrician and medical educator. He has 20 years experience of postgraduate teaching and now teaches clinical skills in the preclinical and clinical years. His research interests include clinical reasoning, curriculum development and teaching clinical skills.
