# Supplementary material for: Using theory to interpret how senior clinicians define, learn, and teach clinical reasoning
Source: MedEdPublish (2016). 2017 Oct 12;6:182. [Version 1] doi: 10.15694/mep.2017.000182 (PMC10885304; doi:10.15694/mep.2017.000182)
Supplement: Peer review history

# Reviewer report: Reviewer response for version 1

**DOI:** 10.21956/mep.19369.r28348

**Published:** 2019-04-13

**Authors:** Ken Masters

**Affiliations:** 1 Sultan Qaboos University

This review has been migrated. The reviewer awarded 3 stars out of 5 An interesting study examining experienced clinicians’ practice of clinical reasoning, and relating it to the theoretical models. The authors have situated their research in the literature, and clearly identified the contribution of this study to the literature.Two issues:• Given the small sample, the exploratory nature of the research, and the fact that the researchers had to use their own non-validated, semi-structured questionnaires, it really would have been better if the researchers had used some sort of saturation methodology in their data-gathering, perhaps more formally using a theoretical model like Grounded Theory. • I do note the rather significant gender imbalance in the sample (12-2), and I wonder if the researchers could have attempted to decrease that balance. Failing that, it would have been useful if the researchers had examined the data in light of this imbalance (i.e. were males more prone to a type of practice/perception, etc. than females?). This would have allowed for some identification of any biases in the results. At the very least, the authors should have mentioned this in their Limitations.Nevertheless, a useful and interesting read.

Reviewer Expertise:

NA

No decision status is available

# Reviewer report: Reviewer response for version 1

**DOI:** 10.21956/mep.19369.r28349

**Published:** 2017-11-09

**Authors:** Gary D. Rogers

**Affiliations:** 1 Griffith University

This review has been migrated. The reviewer awarded 4 stars out of 5 This is a well-written paper that reports the findings of a qualitative study based on interviews with expert clinical educators at one medical school. It focuses on their experience of the practice, teaching and learning of clinical reasoning (CR), analysing their responses first inductively and then interrogating the extent to which they accord with prominent theories of clinical reasoning, principally script theory and dual process theory.The study appears to have been carefully conducted, using sound qualitative methods that were fully described and referenced. Its findings appear highly credible and carefully discussed, with appropriate acknowledgement of the limitations of the study. The introductory literature review is comprehensive but concise and would be very useful for a health professional educator delving into this area for the first time.There are some minor organisational and typographic issues, such as one of the headings (‘how senior clinicians learned clinical reasoning and maintain their skills’) not appearing in heading typeface; one of the quotes (begins ‘I didn’t know I was developing my teaching …’) appearing under this heading when it seems to relate more to the following concept of *teaching* CR; ‘illness scrips’ instead of ‘illness scripts’; ‘reminder’ instead of ‘remainder’; and some references cited in the text (eg Thomas, 2006) not appearing in the reference list. This otherwise excellent paper would benefit from correction of these small issues in a second draft. This is high quality research that adds significantly to our understanding of an important topic.

Reviewer Expertise:

NA

No decision status is available
